# Fasciitis of the Breast, Ranging from Necrotizing to Non-Necrotizing Forms: A Diagnostic Dilemma Necessitating Vigilant Clinical Scrutiny

**DOI:** 10.12669/pjms.42.(11AASC).15806

**Published:** 2026-04

**Authors:** Safna Naozer Virji, Sahra Mateen, Alisha Fatima Rizvi, Lubna Mushtaque Vohra

**Affiliations:** 1 Dr. Safna Naozer Virji, FCPS. Department of Surgery, Aga Khan University Hospital, Stadium Road, Karachi, Pakistan; 2 Sahar Mateen, Medical College, Aga Khan University, Stadium Road, Karachi, Pakistan; 3 Dr. Alisha Fatima Rizvi, FCPS. Department of Surgery, Aga Khan University Hospital, Stadium Road, Karachi, Pakistan; 4 Dr. Lubna Mushtaque Vohra, FCPS. Department of Surgery, Aga Khan University Hospital, Stadium Road, Karachi, Pakistan

**Keywords:** Breast infections, Necrotizing soft tissue infection, Mastectomy

## Abstract

Necrotizing soft tissue infections (NSTIs) are rare but highly aggressive infections characterized by rapid necrosis of the skin, subcutaneous tissue, and fascia. While typically seen in the abdomen, perineum, and extremities, NSTIs of the breast are uncommon and often misdiagnosed as cellulitis, mastitis, or abscess, contributing to delayed treatment and high morbidity and mortality.

In this series, four patients presented with varying severity of breast NSTIs. Two diabetic patients had delayed presentations and required mastectomy, among whom one succumbed to overwhelming sepsis and multi-organ failure despite aggressive debridement and systemic antimicrobial treatment. A third patient with acute viral infection developed extensive chest wall inflammation concerning for necrotizing myositis requiring surgical exploration and debridement. The last case is of a lactating woman, who presented early and responded well to antibiotics alone and was spared the need for surgical intervention.

These cases highlight the broad clinical spectrum of breast NSTIs and emphasize that early recognition and prompt antibiotic therapy with timely surgical debridement are critical for reducing morbidity and mortality of this rare yet aggressive disease in the breast.

## INTRODUCTION

Necrotizing soft tissue infections (NSTIs) are relatively rare but highly aggressive infections with the potential to cause progressive necrosis of the skin, subcutaneous tissue and superficial fascia. Most involve the abdomen, perineum or extremities, with a reported incidence of 0.3 to 15 cases per 100,000.^1^,^2^

NSTIs are usually a result of traumatic inoculation; with diabetics and immunosuppressed individuals being more susceptible to overwhelming infection.^3^ Clinical presentations of NSTIs include localized inflammation and tenderness that are often mistaken for cellulitis, thus delaying diagnosis.^3^

NSTIs of the breast are an even rarer occurrence, with a mortality rate reported as high as 73%.^4^ The onset of these infections is usually preceded by an episode of trauma or surgical intervention and can often times be misdiagnosed as an abscess or cellulitis. This causes a delay in treatment, which possibly contributes to the high mortality rate.^5^ Management of NSTIs is typically done with surgical debridement and administration of antibiotics. A total mastectomy may be required in cases that are diagnosed late; however, cases that are diagnosed earlier may be managed conservatively or with debridement alone.^2^

## CASE PRESENTATIONS

### CASE-1:

A 64 years old diabetic (HbA1c 7.8%) female presented to the emergency room with the complaint of right mastalgia, fever and redness over the breast for three days. She had no history of trauma to the breast. On examination she appeared dehydrated but was vitally stable. The right breast examination showed focal oedema and erythema in the outer half of the right breast with tenderness on palpation. Her workup performed in the emergency department revealed a normal total leukocyte count (TLC) of 5.8x109/L (91.6% neutrophils), a C-reactive protein (CRP) of 340 mg/L and an elevated creatinine of 2.2 mg/dL. The right breast ultrasound reported significant soft tissue edema, likely secondary to mastitis, with multiple tiny fluid pockets.

**Fig.1:**
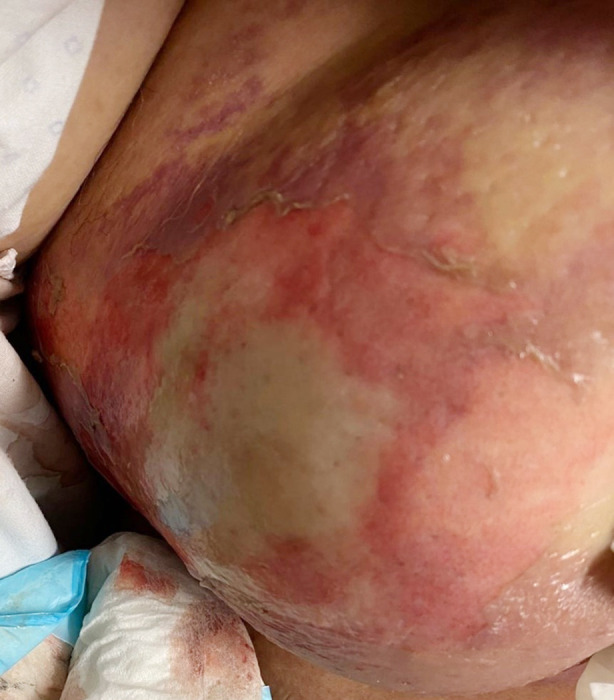
Right breast edema with development of necrotic patch and crepitus on lateral half.

Initial resuscitation commenced with intravenous (IV) fluids and antibiotics. However, her condition continued to deteriorate over the next six hours. She became tachycardic, tachypnoeic and hypotensive with decreased urine output. On re-examination, the right breast had developed a necrotic patch in the upper outer quadrant (UOQ) with significant edema, erythema and crepitus. (Fig.1) She developed metabolic acidosis with a lactate of 12.0 mmol/L. The diagnosis of necrotizing fasciitis of the breast was made, and she underwent urgent right radical mastectomy with extensive debridement. Intra-operatively, there was extensive necrosis of the right breast involving the axilla, right chest wall, right anterior abdomen and right posterior chest wall.

She was shifted to the surgical intensive care unit (ICU), reintubated, and required dual ionotropic support. Antibiotics were escalated to meropenem, clindamycin and vancomycin, and her final tissue cultures reported beta-haemolytic Group A Streptococcus. Daily wound dressings were performed. The patient’s condition, however, continued to deteriorate, and on the fourth postoperative day, she underwent cardiopulmonary arrest and expired.

### CASE-2:

**Fig.2:**
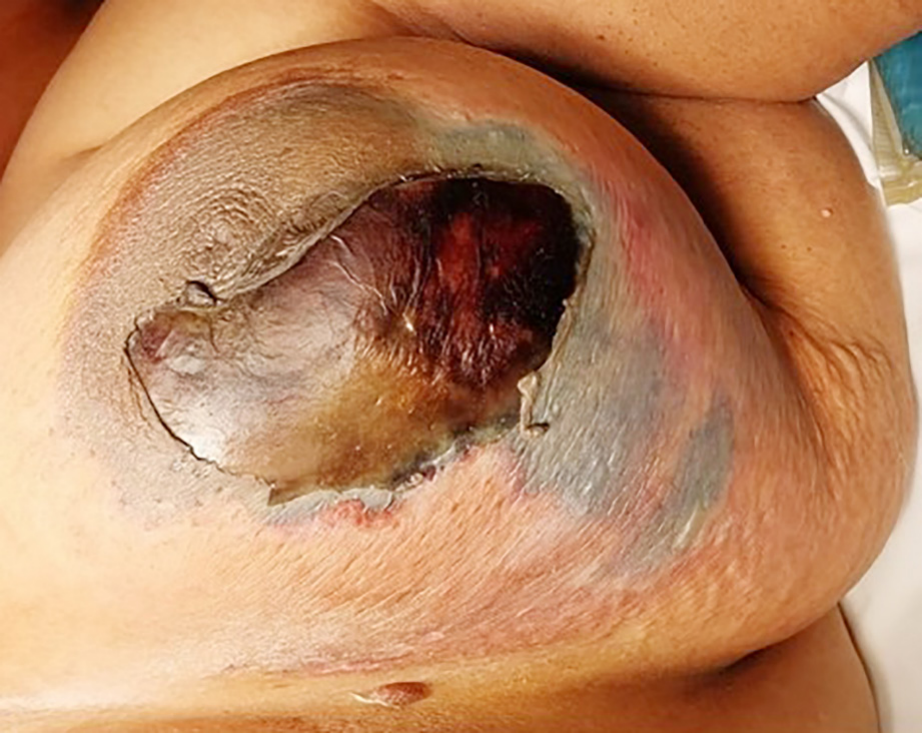
Left breast necrotic patch with de-squamation involving the areola and extending laterally.

A 48 years old female with uncontrolled diabetes (HbA1c 11.9%) presented with the complaint of pain and swelling of the left breast for eight days. She was tachycardic, and the local examination showed left breast erythema and a necrotic patch at 3 o’clock. (Fig.2) Her laboratory results were significant for a TLC of 28.4x109/L (neutrophilic shift 88.1%).

She was diagnosed with NSTI of the left breast and underwent emergent surgery. Intra-operatively, there was extensive necrotic tissue reaching the left pectoralis major and the latissimus dorsi muscles for which she underwent a left mastectomy.

Cultures grew Staphylococcus species (not aureus) and few colonies of Streptococcus species. She was initially managed in the ICU, and as her condition improved, she was shifted out and discharged on the fifth postoperative day on daily wound dressing. A month after her initial surgery, she underwent left chest wall debridement and closure of the mastectomy wound.

### CASE-3:

A 42 years old female presented with the complaint of fever and left mastalgia for 10 days. She was vitally stable, except for intermittent fever spikes of 102°F. On examination there was left lateral chest wall and breast oedema, extending to the left axilla. No erythema, lump or fluctuance was noted on examination. She had a TLC of 24.6x109/L (no neutrophilic shift), a CRP of 67 mg/L and positive Dengue IgM Ab. The left breast ultrasound showed increased skin thickening of 4 mm with underlying minimal edema in the UOQ of the breast. Chest x-ray revealed left pleural effusion.

Initially managed with IV antibiotics, there was no clinical improvement, and the patient continued to complain of significant pain and fever. She had non-necrotising fasciitis; hence, she never developed any skin changes and was difficult to diagnose if not for the MRI, which showed extensive inflammatory changes in the left breast, axilla and chest wall with micro-abscesses and inflammatory changes in the lungs with effusion. She underwent exploration and debridement and video-assisted thoracoscopy (VATS).

Intraoperatively there was necrotic material with 300-400 ml of haemic fluid drained from the axilla. Breast tissue was edematous but otherwise unremarkable. The pectoralis major, latissimus dorsi and subscapularis muscles were oedematous but healthy with reactionary fluid in intermuscular planes. Thoracoscopy revealed 400 ml of straw-coloured pleural effusion, and a chest tube was inserted.

The final histopathology reported fibroadipose tissue showing sheets of degenerated, inflammatory cells along with abscess formation. There was no organism identified on Gram stain or tissue culture. Daily wound dressing was done, and the patient was discharged on the second postoperative day in a stable condition.

### CASE-4:

A 27 years old lactating female presented to the emergency room complaining of right mastalgia and erythema for one day. There was a tender, erythematous patch in the UOQ of the right breast. Her TLC was 15.0x109/L with no neutrophilic shift. The right breast ultrasound showed subcutaneous soft tissue oedema in the UOQ with fluid tracking along the inter-fascial planes.

She was admitted and managed along the lines of mastitis with a close eye for possible necrotizing infection. She was administered IV ceftriaxone and clindamycin, to which she showed symptomatic improvement, and her TLC count decreased. Her pain and erythema improved, and she was discharged on the second day of admission on antibiotics.

## DISCUSSION

The development of NSTI of the breast is extremely rare, with the abdomen and perineum being more commonly involved.^5^ The disease progresses rapidly and can lead to severe morbidity and mortality. Diagnostic delays, often due to its resemblance to other breast pathologies such as cellulitis, mastitis, or abscess, further worsen outcomes.^4^

As demonstrated in the above cases, NSTIs of the breast exist on a spectrum; however, a consistent determinant of improved prognosis is early identification and timely intervention.^6^ In Case Four, early presentation, high clinical suspicion, and prompt initiation of antibiotics prevented the need for surgical debridement. In contrast, patients who presented after more than a week of symptoms required operative management with surgical morbidity and even mortality, as seen in Case One.

Another important factor is the patient’s immune status. Although trauma is typically considered the inciting cause of NSTIs, it is often minor, goes unnoticed, or is not recalled by the patient.^7^ Diabetes mellitus, lactation, and immunocompromised states are known risk factors and are associated with more aggressive disease and poorer outcomes, as reflected in our series.^4^,^7^,^8^ Even rarer are reports of NSTI following systemic viral infections such as COVID-19.^9^ Case three is particularly notable, as the patient developed NSTI while affected by dengue fever, supporting the association between immune compromise and disease susceptibility.

Breast ultrasound (typically the first-line modality), as well as cross-sectional imaging such as CT and MRI, may prove invaluable for early recognition of NSTIs, particularly before overt clinical signs such as crepitus or bullae develop.^10^ The extent of debridement depends on the degree of necrosis, ranging from limited excision of devitalized tissue to mastectomy in severe cases. Despite the potential for significant disfigurement, survival remains the overriding priority.^4^

## CONCLUSION

NSTIs of the breast is a rare presentation, and maintaining a high index of suspicion for its timely diagnosis is essential. Early identification and the prompt initiation of antibiotics and surgical intervention remain the most important determinants of survival. Increased clinical awareness, a heightened degree of suspicion, and judicious use of timely cross-sectional imaging may improve outcomes by facilitating rapid recognition of this uncommon but life-threatening condition.

### Authors’ Contribution:

**SNV**: Data collection, writing and editing of the manuscript.

**SM** and **SAF**: Manuscript writing.

**LMV** conceived, reviewed, edited the manuscript, provided final approval and is responsible for integrity of research.
